# CD68 and interleukin 13, prospective immune markers for esophageal squamous cell carcinoma prognosis prediction

**DOI:** 10.18632/oncotarget.6900

**Published:** 2016-01-12

**Authors:** Jian Li, Bao-Zhu Zhang, Yan-Ru Qin, Jiong Bi, Hai-Bo Liu, Yan Li, Mu-Yan Cai, Stephanie Ma, Kwok Wah Chan, Dan Xie, Xin-Yuan Guan

**Affiliations:** ^1^ State Key Laboratory of Oncology in Southern China, Sun Yat-Sen University Cancer Center, Guangzhou, China; ^2^ Guangdong Provincial Key Laboratory of Malignant Tumor Epigenetics and Gene Regulation, Sun Yat-Sen Memorial Hospital, Sun Yat-Sen University, Guangzhou, China; ^3^ Department of Clinical Oncology, The First Affiliated Hospital, Zhengzhou University, Zhengzhou, China; ^4^ Department of Surgery, The First Affiliated Hospital of Sun Yat-Sen University, Guangzhou, China; ^5^ Key Laboratory for Major Obstetric Diseases of Guangdong Province, The Third Affiliated Hospital of Guangzhou Medical University, Guangzhou Medical University, Guangzhou, China; ^6^ Department of Pathology, Sun Yat-Sen University Cancer Center, Sun Yat-Sen University, Guangzhou, China; ^7^ Department of Anatomy, Li Ka Shing Faculty of Medicine, The University of Hong Kong, Hong Kong, China; ^8^ Department of Pathology, Li Ka Shing Faculty of Medicine, The University of Hong Kong, Hong Kong, China; ^9^ Department of Clinical Oncology, Li Ka Shing Faculty of Medicine, The University of Hong Kong, Hong Kong, China

**Keywords:** ESCC, IL-13, macrophage, prognosis, CD68

## Abstract

**Purpose:**

Oncology immunity was reported to play a key role in cancer development and progression, so we investigated the prediction role of several immune markers in esophageal squamous cell carcinoma (ESCC) patients after operation in this study.

**Patients and Methods:**

66 primary ESCC tumor tissues and four sets of tissue microarrays including 705 primary ESCC tumor tissues from four centers were collected and analyzed. Expressions of several immune markers in ESCC tumor tissue were detected with immunohistochemistry staining. Their distribution densities were analyzed with InForm^™^ 2.0.1 software. All statistic analyses were performed with SPSS16.0 and Stata version 10.0.

**Results:**

Survival analyses assessed by Kaplan-Meier plots and log-rank tests demonstrated that densities of CD68 and interleukin 13 (IL-13) in tumor stroma were positively correlated with the overall survival of ESCC patients after operation (*p* < 0.01 for CD68, *p* < 0.001 for IL-13). Further, a model based on tumor stroma densities of CD68 and IL-13 was constructed and it could significantly classify patients with poor or good prognosis. This model could further identify high-risk group and low-risk group at the same Tumor lymph Nodes Metastases (TNM) stage. Lastly, a more accuracy model based on TNM stage, densities of CD68 and IL-13 was constructed to predict the prognosis of ESCC patient after operation.

**Conclusion:**

Combining the TNM staging system and densities of CD68 and IL-13 could substantially improve the prognosis prediction accuracy of ESCC patient after operation, which might be an excellent tool for selecting patients for individualized therapy in future.

## INTRODUCTION

It is estimated that there were 482,300 new esophageal squamous cell carcinoma (ESCC) cases and 406,800 deaths in 2008 worldwide [1]. ESCC incidence varies as high as 16-fold in different areas [1]. Current Tumor lymph Nodes Metastases (TNM) staging system provides a useful benchmark for ESCC prognosis estimation and treatment strategy establishment. This system, however, is only based on the anatomical extent (tumor, lymph node and distant metastasis), histopathology type, biological activity and tumor location, which does not include the immune state of patient [2]. The role of immune system in cancer development and progression has been well established both in mice model [3, 4] and on clinic [5, 6]. ESCC was not excluded from this dogma. Many immune cells and their related factors had been reported to be involved in the progression of it [7–9]. It is highly possible that the immune status of ESCC patient may provide useful information for the prognosis prediction, which may help to explain the discrepancy prognosis at the same TNM stage.

Rapid progress of immunology unveils plenty of immune specific markers, which aids to the prompt detection of patient's immune status with immunohistochemistry (IHC) staining [10, 11]. We screened the expression and distribution of a panel of immune markers in ESCC primary tumor samples firstly, and found that the distribution of CD68 and interleukin 13 (IL-13) could distinguish between good and poor prognosis. We then constructed a model based on these two immune markers to predict ESCC patients’ prognosis, which was further validated with samples from three external centers. This model could also classify high risk and low risk group even at the same TNM stage. A model based on TNM stage, densities of CD68 and IL-13 was further constructed to predict the prognosis of ESCC patient after operation.

## RESULTS

### Distribution features of different immune markers in ESCC tumor area and adjacent area

**Figure 1:**
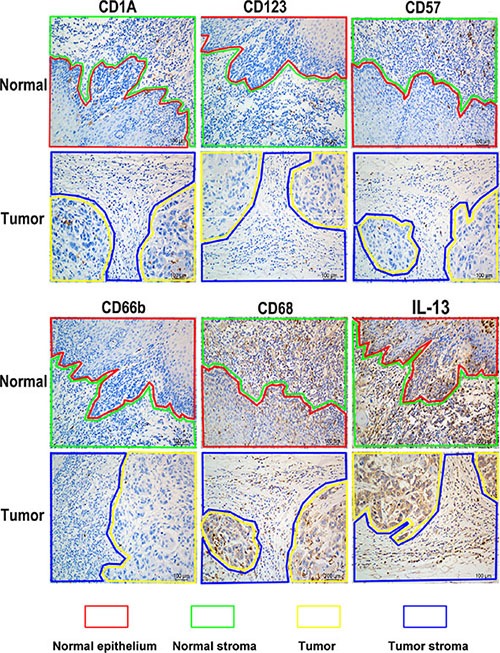
Representative images of different immune markers’ distribution in ESCC tissues detected with IHC staining Lines of different color represented diverse areas of esophagus tissue. Positive stained cells were demonstrated as brown.

Distribution features of CD1A, CD123, CD57, CD66b, CD68 and IL-13 were detected in 20 ESCC patients’ tumor tissues with IHC staining. Results demonstrated that they distributed differently in diversity areas (Figure 1). CD1A positive cells mainly distributed in tumor and normal stroma around the normal epithelium. CD123 positive cells mainly distributed in tumor stroma and normal stroma. CD57 and CD66b positive cells mainly existed in normal stroma and tumor stroma as well as in tumor area sometimes. CD68 and IL-13 positive cells distributed exclusively but with different densities in different areas. Higher magnification demonstrated the cellular location of respective marker was also provided (Supplementary Figures 1–6). From results of preliminary experiments, we observed that positive cells of CD1A, CD68 or IL-13 had distinct distribution among ESCC patients with good or poor prognosis.

### Densities of CD68 and IL-13 positive cells in tumor stroma correlated to the survival time of ESCC patient after operation

**Figure 2:**
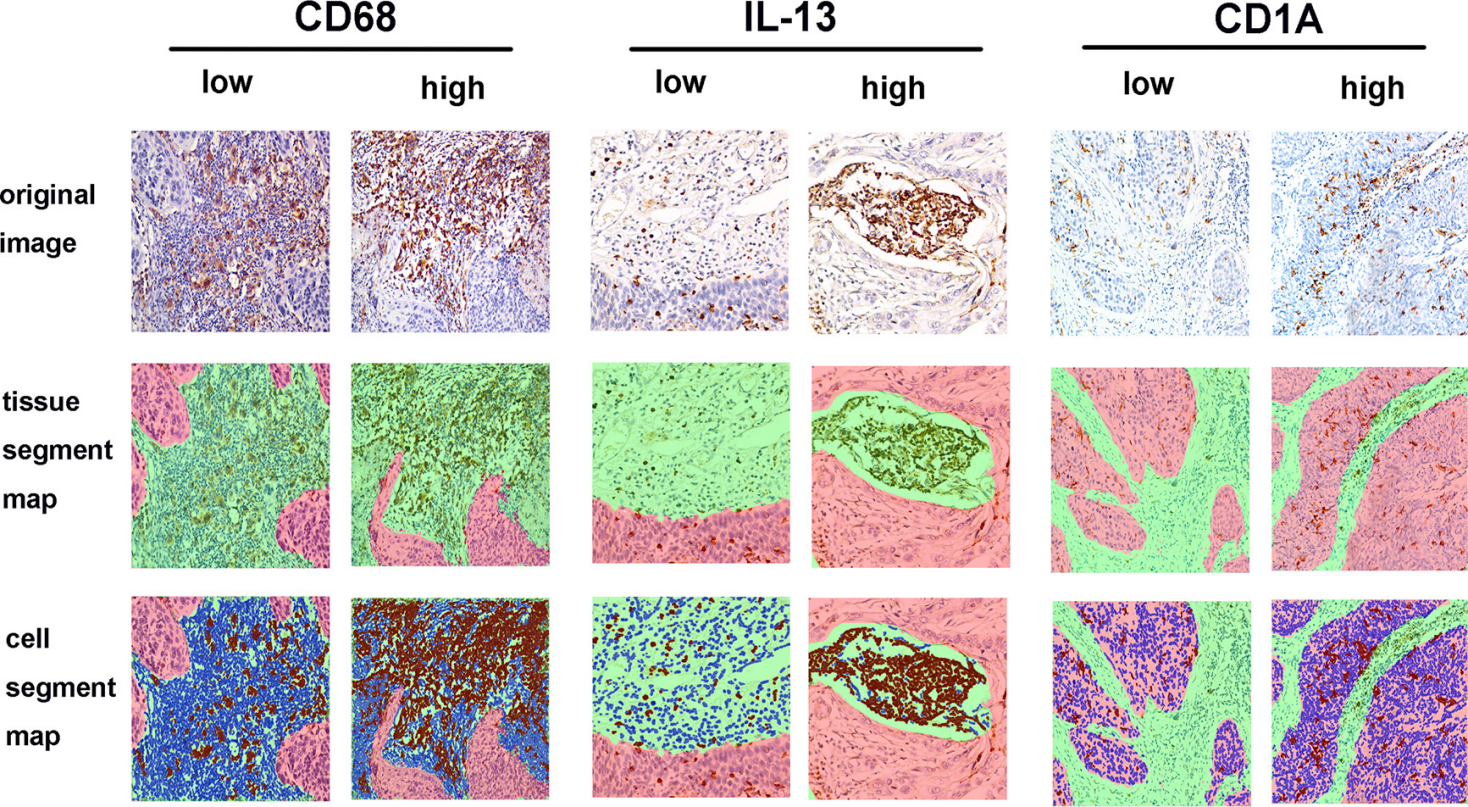
Diagrammatic sketch of immune markers’ distribution density in different areas processed by InForm^™^ 2.0.1 software The upper row presented original images that represented two different expression patterns (low density and high density) of CD68, IL-13 and CD1A. The middle row demonstrated segment maps, which divided tissues into tumor area (red) and tumor stroma area (green) processed by InForm^™^ 2.0.1 software. In bottom row, the nucleus of tumor cells or tumor stroma cells are marked in blue, Positive stained cells in tumor or tumor stroma were marked in brown.

**Figure 3:**
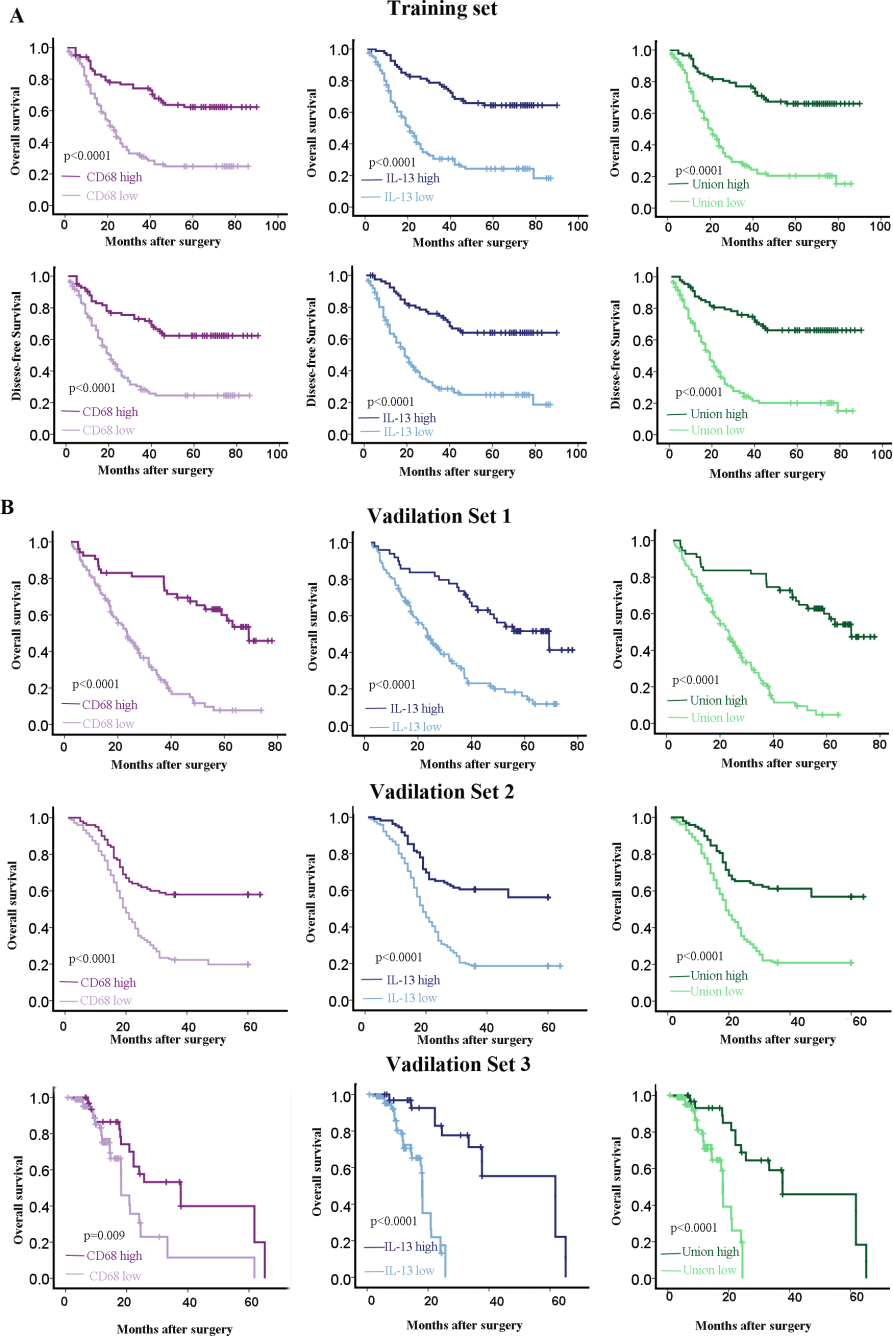
Prognosis prediction of CD68, IL-13 and union model in ESCC patients (**A**) Kaplan-Meier analysis demonstrated that high density of CD68 or IL-13 or union had good overall survival and disease-free survival for ESCC patient from Sun Yat-Sen University cancer center (training set). (**B**) Kaplan-Meier analysis validated that high density of CD68 or IL-13 or union had good overall survival for ESCC patients from three external centers. Validation set 1, The First Affiliated Hospital, Sun Yat-sen University; validation set 2, Linzhou Cancer Hospital (Henan, China); validation set 3, Hong Kong University.

Distribution densities of CD1A, IL-13 and CD68 in tumor stroma of 66 ESCC patients were further calculated with InForm™ 2.0.1 software (Figure 2). Results demonstrated that densities of CD68 and IL-13 in tumor stroma area were probably associated with the prognosis of patients. So the correlation between density of CD68 as well as IL-13 in tumor stroma and patients’ survival time was further confirmed by a TMA containing 194 ESCC tumor samples from the same center (training set). ROC curves analyses showed the optimum cutoff value for density of CD68 was 87 (CD68 density ≥ 87 was defined as CD68^high^ group, CD68 density <87 was defined as CD68^low^ group) and IL-13 was 23 (IL-23 density ≥ 23 was defined as IL-13^high^ group, IL-23 density < 23 was defined as IL-13^low^ group) respectively. Survival analyses assessed by Kaplan-Meier plots and log-rank tests disclosed that densities of both CD68 and IL-13 in tumor stroma were significantly positively correlated to the overall survival time and disease-free survival time of patients after operation (*p* < 0.0001, Figure 3A).

### Model based on the tumor stroma densities of CD68 and IL-13 demonstrated priority at the prognosis prediction after operation

Univariate and multivariate Cox regression analysis were performed to explore whether CD68 and IL-13 were independent prognostic factors for ESCC patient or not (Table 1). The result demonstrated that tumor stroma densities of CD68 and IL-13 were independent prognostic factors for patients’ overall survival (*p* < 0.01). To explore whether they had more accuracy prognostic function when combined, we derived a model to calculate the union score for every patient with the densities of them and their weighted regression coefficient.

**Table 1:** Univariate and multivariate cox regression analysis for training set from SunYat-sen university cancer center

Variable	Univariate		Multivariate			
	DFS	OS	DFS		OS	
	*P* value	*P* value	HR (95% CI)	*P* value	HR (95% CI)	*P* value
Gender	0.500	0.053				
Age	0.596	0.904				
Location	0.349	0.438				
Grade	0.054	0.082				
TNMstage	0.014	< 0.001	1.417 (0.985–2.037)	0.060	1.640 (1.172–2.295)	< 0.001
CD68	0.010	< 0.001	0.597 (0.388–0.917)	0.018	0.457 (0.307–0.681)	< 0.001
IL-13	0.039	< 0.001	0.670 (0.438–1.025)	0.065	0.376 (0.250–0.567)	0.004

In this comparison, TNM staging including TNM stage I, II, III and IV was considered as ordinal variable; Gender including male and female as nominal variable; Age including age ≥ 60 and age < 60 was considered as nominal variable; Location, which meant ESCC located in upper chest, middle chest or low chest, was considered as nominal variable; Grade, which meant degree of differentiation including poor-differentiated, moderately differentiated or well-differentiated, was considered as ordinal variable.

Union score = Density of CD68 * 0.711 + Density of IL-13 * 0.632

ROC curves analyses displayed that the optimum cutoff value for union score was 78, which divided patients (*n* = 194) into Union^high^ group (union score ≥ 78) and Union^low^ group (union score < 78) Pearson chi-square test showed that clinicopathological characteristics did not vary significantly between the high-risk and low-risk group (Table 2). Further, the relation of protective score and survival status was analyzed and the result demonstrated that patients with higher union score generally had longer survival time than those with lower score (Figure 3A). 5-year overall survival was 66% (95% CI 60.9–91.1) for the low-risk group, and 20% (95% CI 19.9–20.4) for the high-risk group respectively (HR 0.358, 95% CI 0.249–0.514).

**Table 2:** Clinicopathological characteristics of ESCC patients from different centers

Clinical features	Training set			Validation set 1			Validation set 2			Validation set 3		
	Case	High (%)	Low (%)	Case	High (%)	Low (%)	Case	High (%)	Low (%)	Case	High (%)	Low (%)
**Age (years old)** < 60 ≥ 60	127 67	68 (53.5%) 38 (56.7%)	59 (46.5%) 29 (43.3%)	90 66	65 (72.2%) 46 (70.0%)	25 (28.0%) 20 (30.0%)	137 119	84 (61.3%) 74 (62.2%)	53 (38.7%) 45 (37.8%)	26 53	19 (73.1%) 43 (81.1%)	7 (26.9%) 10 (18.9%)
**Gender** Male Female	106 88	74 (69.8%) 64 (72.7%)	32 (30.2%) 24 (27.3%)	110 46	74 (67.3%) 26 (56.5%)	36 (32.7%) 20 (43.5%)	152 121	106 (69.7%) 88 (72.7%)	46 (30.3%) 33 (27.3%)	62 17	49 (79.0%) 13 (76.5%)	13 (21.0%) 4 (23.5%)
**Location** Upper Middle Lower	15 141 38	9 (60.0%) 79 (56.0%) 18 (47.4%)	6 (40.4%) 62 (44.0%) 20 (52.6%)				53 171 20	41 (77.4%) 97 (56.7%) 13 (65.0%)	12 (22.6%) 74 (43.3%) 7 (35.0%)			
**Differentiation** Grade 1 Grade 2 Grade 3	17 131 46	3 (17.6%) 71 (54.2%) 32 (69.6%)	14 (82.4%) 60 (45.8%) 14 (30.4%)	28 74 39	23 (82.1%) 45 (60.8%) 27 (69.2%)	5 (17.9%) 29 (39.2%) 12 (30.8%)	23 164 64	15 (65.2%) 94 (57.3%) 44 (68.8%)	8 (34.8%) 70 (42.7%) 20 (31.2%)	16 43 20	13 (81.2%) 32 (74.4%) 17 (78.5%)	3 (18.8%) 11 (25.6%) 3 (21.5%)
**pN category** N0 N1	104 90	51 (49.0%) 55 (61.1%)	53 (51.0%) 35 (38.9%)	106 50	63 (78.6%) 37 (61.3%)	43 (21.4%) 13 (38.7%)	151 105	93 (61.6%) 65 (61.9%)	58 (38.4%) 40 (38.1%)	29 50	24 (82.8%) 38 (76.0%)	5 (17.2%) 12 (24.0%)
**TNM stage** Early (I–II) Advance (III–IV)	117 76	56 (47.9%) 49 (64.5%)	61 (52.1%) 27 (35.5%)	125 31	73 (58.4%) 27 (87.1%)	52 (41.6%) 4 (12.9%)	184 89	142 (56.3%) 52 (35.5%)	42 (43.6%) 37 (64.5%)	32 47	23 (71.9%) 39 (83.0%)	9 (28.1%) 8 (17.0%)

All 705 patients had undergone surgical resection and none of them received preoperative treatment. The median follow-up was 23 months (IQR 13–36). Training set, Sun Yat-sen University Cancer Center; validation set 1, The First Affiliated Hospital, Sun Yat-sen University; validation set 2, Linzhou Cancer Hospital (Henan, China); validation set 3, Hong Kong university.

### Prognosis prediction of CD68 and IL-13 based model was validated from other three centers

Another 511 ESCC patients tumor tissues from three different centers including Northern and Southern areas of China (validation sets) (Table 2) were collected to confirm whether CD68 and IL-13 based model had popular prognostic priority in patients from different areas or not. As only overall survival data were collected in these 511 cases, we analyzed only the overall survival prognosis for them (Table 3). Kaplan-Meier analysis indicated that CD68 or IL-13 or union score with high value was associated with good prognosis and long overall survival time (Figure 3B). We observed similar relation of protective scores and survival status in the training set (HR 0.358, 95% CI 0.249–0.514; *p* < 0.0001; Table 3), as well as in three independent validation sets: validation set 1 (HR 0.247, 95% CI 0.160–0.382; *p* < 0.0001; Table 3), validation set2 (HR 0.186, 95% CI 0.112–0.308; *p* < 0.0001; Table 3) and validation set 3 (HR 0.206, 95% CI 0.085–0.498; *p* < 0.0001; Table 3).

**Table 3:** Univariate cox regression analysis of overall survival with different parameters in four centers

	Training set		Validation set 1		Validation set 2		Validation set 3	
	HR(95%CI)	*p* value	HR(95%CI)	*p* value	HR(95%CI)	*p* value	HR(95%CI)	*p* value
**Age** (< 60* VS ≥ 60*)	1.286 (0.959–1.725)	0.093	1.025 (0.686–1.531)	0.904	4.995 (3.312–7.535)	< 0.001	1.93 (1.07–3.484)	0.029
**Gender** **(male VS female)**	0.881 (0.654–1.187)	0.406	0.612 (0.372–1.006)	0.053	0.899 (0.585–1.380)	0.626	0.482 (0.241–0.962)	0.039
**Differentiation** **(poor VS well)**	1.344 (1.072–1.685)	0.01	1.355 (0.962–1.908)	0.082	1.004 (0.743–1.356)	0.981	0.994 (0.667–1.483)	0.978
**Lymphatic metastasis** **(yes VS no)**	2.080 (1.548–2.795)	< 0.001	2.327 (1.579–3.427)	< 0.001	1.77 (1.196–2.620)	0.004	1.091 (0.629–1.892)	0.757
**Distant metastasis** **(yes VS no)**	2.505 (1.025–6.118)	0.044					2.032 (1.186–3.482)	0.01
**TNMstage** **(advanced VS early)**	2.306 (1.726–3.081)	< 0.001	2.423 (1.654–3.551)	< 0.001	2.002 (1.29–3.107)	0.002	1.547 (0.893–2.678)	0.12
**CD68** **(high VS low expression)**	0.559 (0.412–0.758)	< 0.001	0.387 (0.261–0.574)	< 0.001	0.427 (0.284–0.642)	< 0.001	0.31 (0.16–0.602)	0.001
**IL-13** **(high VS low expression)**	0.43 (0.315–0.588)	< 0.001	0.313 (0.211–0.464)	< 0.001	0.437 (0.292–0.654)	< 0.001	0.348 (0.186–0.654)	0.001
**Union** **(high VS low expression)**	0.358 (0.249–0.514)	< 0.001	0.247 (0.16–0.382)	< 0.001	0.186 (0.112–0.308)	< 0.001	0.206 (0.085–0.498)	< 0.001

* Years old. Training set, Sun Yat-sen University Cancer Center; validation set 1, The First Affiliated Hospital, Sun Yat-sen University; validation set 2, Linzhou Cancer Hospital (Henan, China); validation set 3, Hong Kong university.

### CD68 and IL-13 based model was superior to TNM staging system at prognosis prediction in these 705 ESCC patients

**Figure 4:**
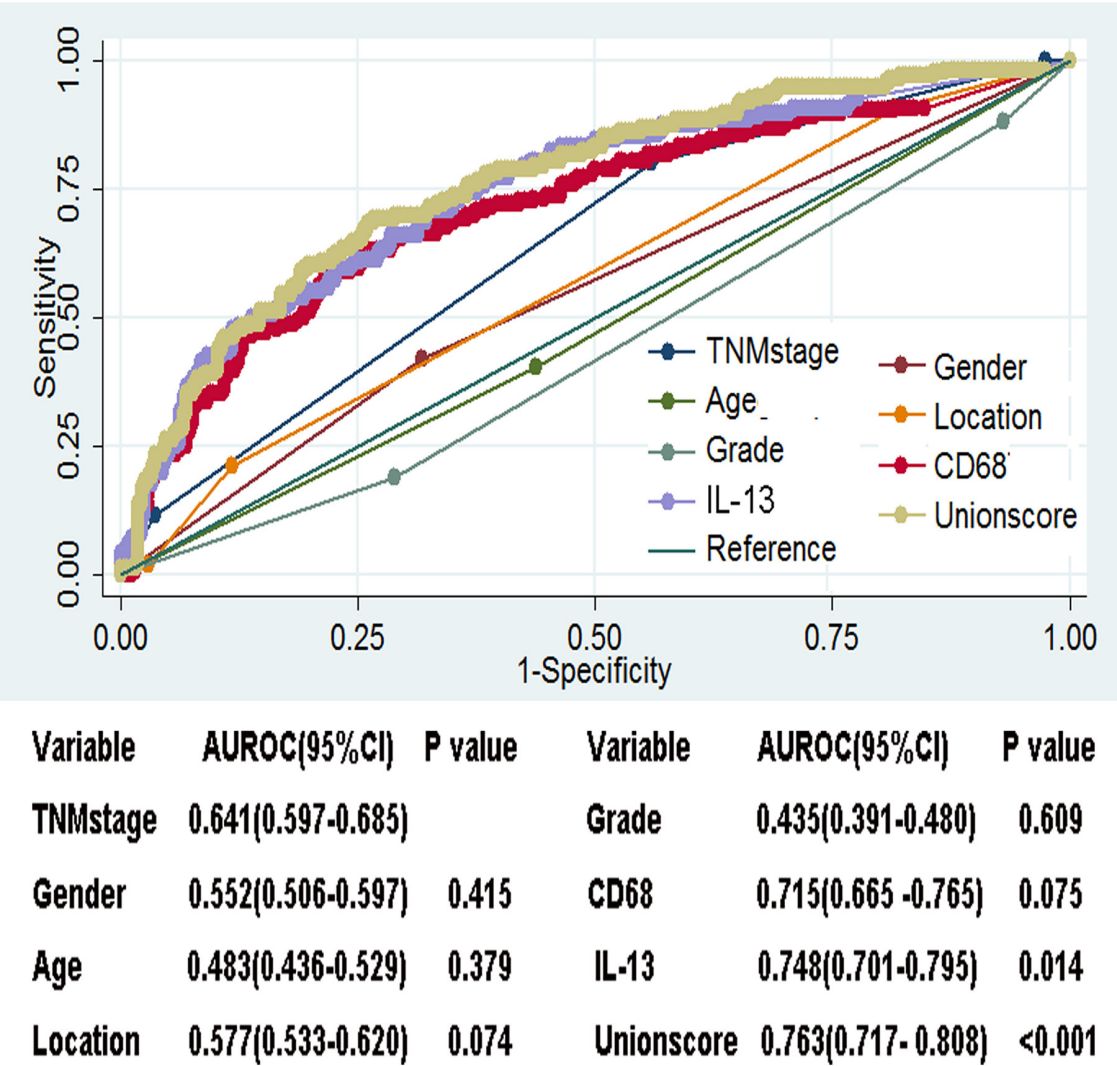
CD68 and IL-13 based model displayed superior prognosis function compared with TNM staging system and other clinicopathological factors as well as single immune marker ROC curves analyses of indicated elements in 705 ESCC patients.

Next we compared the prediction accuracy of the two-immune marker-based model with other clinicopathological risk factors and single immune marker with ROC curves analyses. The result revealed that CD68 and IL-13 density based union model demonstrated significantly higher prognostic accuracy than indicated clinicopathological factors including TNM staging in these 705 ESCC patients (Figure 4). In the analyses, TNM staging including TNM stage I, II, III and IV was considered as ordinal variable. Gender including male and female was considered as nominal variable. Age including age ≥ 60 and age < 60 was considered as nominal variable. Location, which meant ESCC located in upper chest, middle chest or low chest, was considered as nominal variable. Grade, which meant degree of differentiation including poor-differentiated, moderately differentiated, well-differentiated, was considered as ordinal variable.

### CD68 and IL-13 based model could further identify high risk and low risk population at the same TNM stage

**Figure 5:**
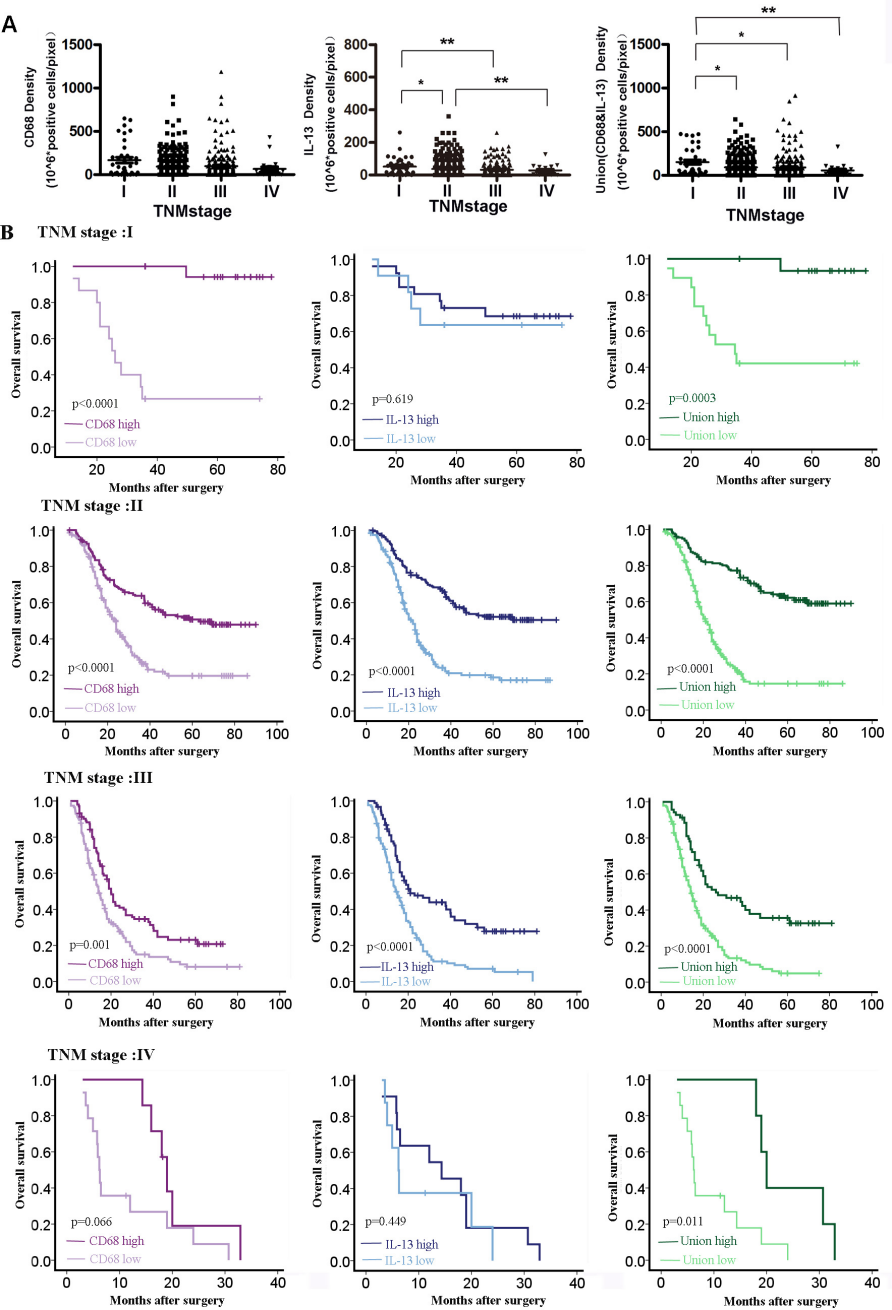
CD68 and IL-13 based model could identify high risk and low risk population at the same TNM stage (**A**) Tumor stroma CD68 density, IL-13 density and union model value at different TNM stages, *P < 0.05; **P < 0.01. (**B**) Prognosis prediction of CD68, IL-13 and union model at different TNM stages for ESCC patients.

We also found that ESCC patient in advanced TNM stage had relatively low tumor stroma IL-13 density and low union score (Figure 5A). Further, we explored the prediction ability of CD68, IL-13 and union model at different TNM stages. Results demonstrated that CD68 and IL-13 density based model could further identify low-risk group and high-risk group at every TNM stage (Figure 5B). Tumor stroma CD68 density could only identify low-risk group and high-risk group at TNM stage I, II and III (Figure 5B). Tumor stroma IL-13 density could only identify low-risk group and high-risk group at TNM stage II and III (Figure 5B).

### Model combining TNM stage, CD68 and IL-13 predicted more accuracy than any others

As CD68 density, IL-13 density and TNM stage were proved to be independent prediction factors for ESCC prognosis evaluated by multivariate cox regression analysis in all cases (*n* = 705) (Table 4), we further explored whether we could make more accuracy prediction or not by combining them. A formula based on the TNM stage, tumor stroma CD68 density, IL-13 density and their respective weighted regression coefficient was further constructed:

**Table 4:** Univariate and multivariate cox regression analysis for all cases

Clinical Features	Univariate analysis		Multivariate analysis	
	HR (95% CI)	*P* value	HR (95% CI)	*P* value
**Gender**	0.899 (0.585–1.380)	0.626		
**Age**	1.004 (0.743–1.356)	0.981		
**Grade**	0.994 (0.667–1.483)	0.978		
**TNMstage**	2.020 (1.748–2.335)	< 0.001	1.959 (1.683–2.281)	< 0.001
**CD68TS**	0.449 (0.369–0.545)	< 0.001	0.542 (0.444–0.662)	< 0.001
**IL-13TS**	0.384 (0.316–0.468)	< 0.001	0.456 (0.372–0.558)	< 0.001

United score = Density of CD68 * 0.003 + Density of IL + 13 * 0.013 – TNM stage * 0.682

**Figure 6:**
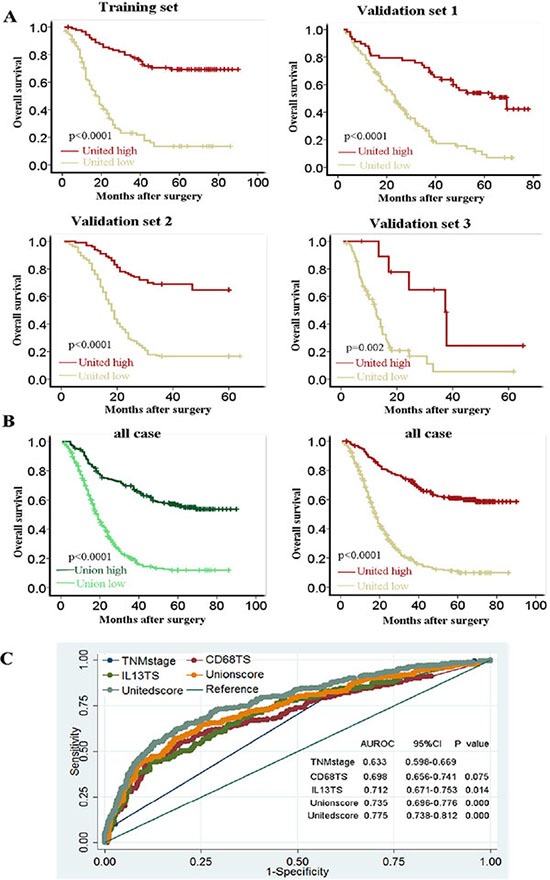
Prognosis prediction of united model compared with TNM stage, single immune marker and two-immune marker based model in survival function, sensitivity and specificity (**A**) Kaplan-Meier analysis demonstrated that ESCC patients with high united score had good overall survival in four different centers. Training set, Sun Yat-sen University Cancer Center; validation set 1, The First Affiliated Hospital, Sun Yat-sen University; validation set 2, Linzhou Cancer Hospital (Henan, China); validation set 3, Hong Kong University. (**B**) Overall survival prediction function comparing of Union and United model in all cases. (**C**) ROC curves of indicated markers.

The value of TNM stage was identified as following, TNM stage I as 1, TNM stage II as 2, TNM stage III as 3, and TNM stage IV as 4. Patients were divided into United^low^ group (United score < 0.82) and United^high^ (United score ≥ 0.82) group with the optimum cutoff value (0.82). Prediction result of united score in different centers was further analyzed with Kaplan-Meier analysis (Figure 6A) as well as in all cases combined (Figure 6B). United score predicted more accuracy than TNM stage or CD68 alone or IL-13alone or union score (Figure 6C).

### Double staining of IL-13 with different immune markers

Double staining of IL-13 with CD3, or CD56, or CD68, or CD20, or CD4 or CD8 was further performed to explore the source of IL-13 in ESCC patients. Results demonstrated that IL-13 could be expressed by CD3, CD4, CD8, CD56, CD68 and CD20 positive cell respectively in ESCC tumor tissue (Supplementary Figure 7).

## DISCUSSION

Prognosis prediction played a critical role in the determination of therapy strategy for cancer patients. With the development of biology and new technique, many factors were reported to be correlated with the progression and prognosis of ESCC. [12–16] However, the TNM staging system is still the most popular accepted prognosis estimation for ESCC patients in clinical practice. Cancer is a system disease that plenty of elements were involved, so the ideal prognosis prediction model of which should be based on the integration analysis of all elements. It is inevitable to observe large variations in the clinical outcome of ESCC patient even with the same TNM stage, as the TNM staging system for ESCC mainly based on the anatomical extent (tumor, lymph node and distant metastasis), histopathologic type, biological activity and tumor location [2].

The immune system plays a key role in the progression of cancer. According to the description of Robert D. Schreiber et al., the correlation between immune system and cancer can be divided into three different phases [17]. In the full landscape of cancer development, the immune system played different roles. It could not only prevent the development of tumor by T cells, natural killer cells and natural killer T (NKT) cells secreted cytotoxic elements such as interferon-alpha (IFN-a), granzyme, perforin et al. [18–22]. It could promote the progression of tumor by tumor associated macrophages, myeloid-derived suppressor cells and regulatory T cells [23, 24]. The immune system was ranked as the emerging hallmark for cancer, because of the important and diverse role it played in tumor progression [25]. So in this study, we investigated the prognosis value of different immune markers in ESCC patients.

We systemically searched Pub Med for reports published before Feb 10, 2011 with the terms “esophageal squamous cell carcinoma”, “immune” and “prognosis”, and found that several immune markers were reported to be correlated with the prognosis of ESCC patients. While no systemically study was reported and evaluated from other centers and applied to clinics. We planned to investigate the prognosis role of immune markers systemically and profoundly. So we began with investigating the expression and distribution of 21 different immune markers with different functions in tumor immunity to screen the possible correlation of their expressions with ESCC patients’ prognosis firstly. Six of them, as we reported in the manuscript, were selected for further study according to their expression characteristics after the first screen. We discovered that different immune markers distributed differently, even in the same patient, which might be correlated with the diversity of the immune system and the distinctive function of respective immune cell or cytokine. We further found that distribution densities of CD68 and IL-13 in tumor stroma area were positively correlated with ESCC patients’ overall survival after operation. CD68 was a popular marker for macrophages, which had been reported by plenty of investigations. The function of macrophages varied with local microenvironment and tumor characteristics. [25] For example, macrophages were reported to be associated with tumor progression and poor prognosis in breast cancer, [26] classic Hodgkin's lymphoma, [27] and hepatocellular cancer, [28] et al. High macrophage density was also reported to be correlated with increased survival in non-small cell lung cancer [29]. We also found that patients with high density of tumor stroma CD68 positive cells had long overall survival in ESCC especially at TNM stage I, II and III. We also found that high density of IL-13 was a good prognostic factor for ESCC patients in this investigation. IL-13 was a cytokine which was first reported to be secreted by activated type 2 T helper (Th2) cells, with the development of immunity, many innate immune cells were reported to can secrete IL-13 including eosinophils, basophils, mast cells, natural killer cells, NKT cells and group 2 innate lymphoid cells. [30, 31] IL-13 was not only reported to play important role in helminthic parasites infection, allergic asthma, ulcerate colitis and eosinophilic esophagitis, [31, 32] which was reported to prevent tumor development and progression. For example, Low serum IL-13 concentration was reported to be correlated with poor prognosis of colorectal cancer, [33] and IL-13 had been reported to can protect mice from papilloma formation during 9,10-dimethyl-1,2-benzanthracene/12-O-tetradecanoylphorbol-13-acetatetwo-step skin carcinogenesis via IL-4Rα induced signaling pathway. [34] In this investigation, IL-13 was found to be high expressed at the early stage of ESCC patient and high IL-13 density in tumor stroma predicted good prognosis, so IL-13 might also play similar protective role in ESCC progression.

Further, we developed a model based on densities of CD68 and IL-13, which was superior to the TNM stage system in the prognosis prediction of ESCC in the analyzed 705 ESCC patients. It implied the important function of immune system in ESCC progression from another way. As TNM staging system was a comprehensive staging system, which had been evaluated by thousands of patients on clinic, the priority of CD68 and IL-13 based model to TNM staging system in ESCC prognosis prediction required further evaluation on clinic. The two immune markers based model could further identify high and low risk ESCC population even at the same TNM stage, which could provide more useful information for personalized therapy. So we further combined TNM stage and densities of CD68 and IL-13 in tumor stroma to construct a united model for ESCC prognosis prediction, which proved to can provide more accuracy prognosis prediction for ESCC patients after operation. As sample in our investigation was acquired from four different centers including ESCC high prevalence area such as Linzhou in North China as well as normal ESCC prevalence area as Guangzhou and Hong Kong in South China, the investigation could provide popular property for ESCC patients.

## MATERIALS AND METHODS

### Specimen and sample

66 ESCC primary tumor specimens were applied for initial observation. Four sets of tissue microarrays (TMA) that contained formalin-fixed paraffin-embedded tumor tissues from 705 ESCC patients were performed in this study. TMA containing 194 primary ESCC tumor samples from Sun Yat-sen University cancer center (Guangzhou, China) was used as the training set, TMAs for validation were obtained from the First Affiliated Hospital, Sun Yat-sen University (Guangzhou, China, *n* = 156), Linzhou Cancer Hospital (Henan, China, *n* = 274) and Hong Kong University (Hong Kong, China, *n* = 81) respectively. None of these patients received preoperative treatment. Samples collected in this study were obtained under the approval of the Committees for Ethical Review of Research involving Human Subjects of Zhengzhou University, Sun Yat-sen University and Hong Kong University. TMA was constructed as described previously. [35]

### IHC staining

Paraffin-embedded, formalin fixed tissues and TMA sections were deparaffinized and nonspecific bindings were blocked with 5% BSA in PBS for 30 mins, RT. Tissues were then incubated with first antibody against human CD1A (Dako, Denmark), or CD123 (Zhongshan Goldengridge, China), or CD57 (Zeta Cooperation, Sierra Madre, CA), or CD66b (Becton Dicknson, Bedford, MA) or CD68 (Abcam, UK), or IL-13 (Boster, China) at 4°C overnight, and subsequently incubated with horseradish peroxidase (HRP)-conjugated second antibody (Dako, Denmark). Diaminobenzidine tetrahydrochloride (DAB) was used as the visualization substrate followed by counter staining with hematoxylin. Positively stained cells were counted with InForm™ 2.1software under microscope.

For double staining, formalin fixed ESCC tissues were deparaffinized and nonspecific bindings were blocked with 5% BSA in PBS for 30 mins, RT. Tissues were then incubated with first antibody (rabbit origin) against human IL-13 and another first antibody (mouse origin) against human CD3 (Gene Tech Company, China), or CD4 (Gene Tech Company, China), or CD8 (Gene Tech Company, China), or CD20 (Gene Tech Company, China), or CD68, or CD56 (Gene Tech Company, China) at 4°C overnight. Subsequently, tissues were incubated with mixer of HRP-conjugated goat anti-mouse IgG and alkaline phosphatase (AP)-conjugated goat anti-rabbit IgG (Golden Bridge International, USA). After washing, tissues were visualized with DAB and AP-red working solution. Hematoxylin was finally used to stain the nucleus.

### Automatic image acquisition and analysis

Vectra platform (Perkin-Elmer, Waltham, MA) was used for acquiring multispectral images (8bit). Nuance 3.0 software (Perkin-Elmer, Waltham, MA) was then used to build distinctive spectral curves for both of the two chromogens (hematoxylin and DAB), then unmixed the signals of these images. In order to segregate tumor and stroma, 20% of these images were trained with InForm™ 2.0.1software (Perkin-Elmer, Waltham, MA). After training, area of tumor or stroma, numbers of tumor cell, stroma cell or positive stained cell in tumor area and stroma area were acquired with InForm™ 2.0.1software respectively. To calculate more reasonable and reliable, the distribution density of immune marker was calculated as 10^6*number of positive cells divided by area in pixel acquired with InForm™ 2.0.1 software.

### Statistical analysis

All statistical analysis was performed with SPSS16.0 and Stata version 10.0 with two-tailed tests, and significance was defined as *p* values less than 0.05. The overall survival (OS) of each parameter was estimated with Kaplan-Meier method and long-rank test. The hazard ratio (HR) was calculated with multivariate Cox regression analysis. Receiver operating characteristics (ROC) curves were used to compare the sensitivity and specificity for the prediction of survival by immune markers and TNM stage. The relation between clinical characteristics and risk score model value was assessed with Student's *t* test, or χ^2^ Chi-Square test.

## SUPPLEMENTARY MATERIALS FIGURES


