# Supplementary material for: CD68 and interleukin 13, prospective immune markers for esophageal squamous cell carcinoma prognosis prediction
Source: Oncotarget. 2016 Jan 12;7(13):15525–38. doi: 10.18632/oncotarget.6900 (PMC4941258; doi:10.18632/oncotarget.6900)
Supplement: Supplementary file 1 [file oncotarget-07-15525-s001.pdf]

CD68 and interleukin 13, prospective immune markers for esophageal squamous cell carcinoma prognosis prediction

Supplementary Materials

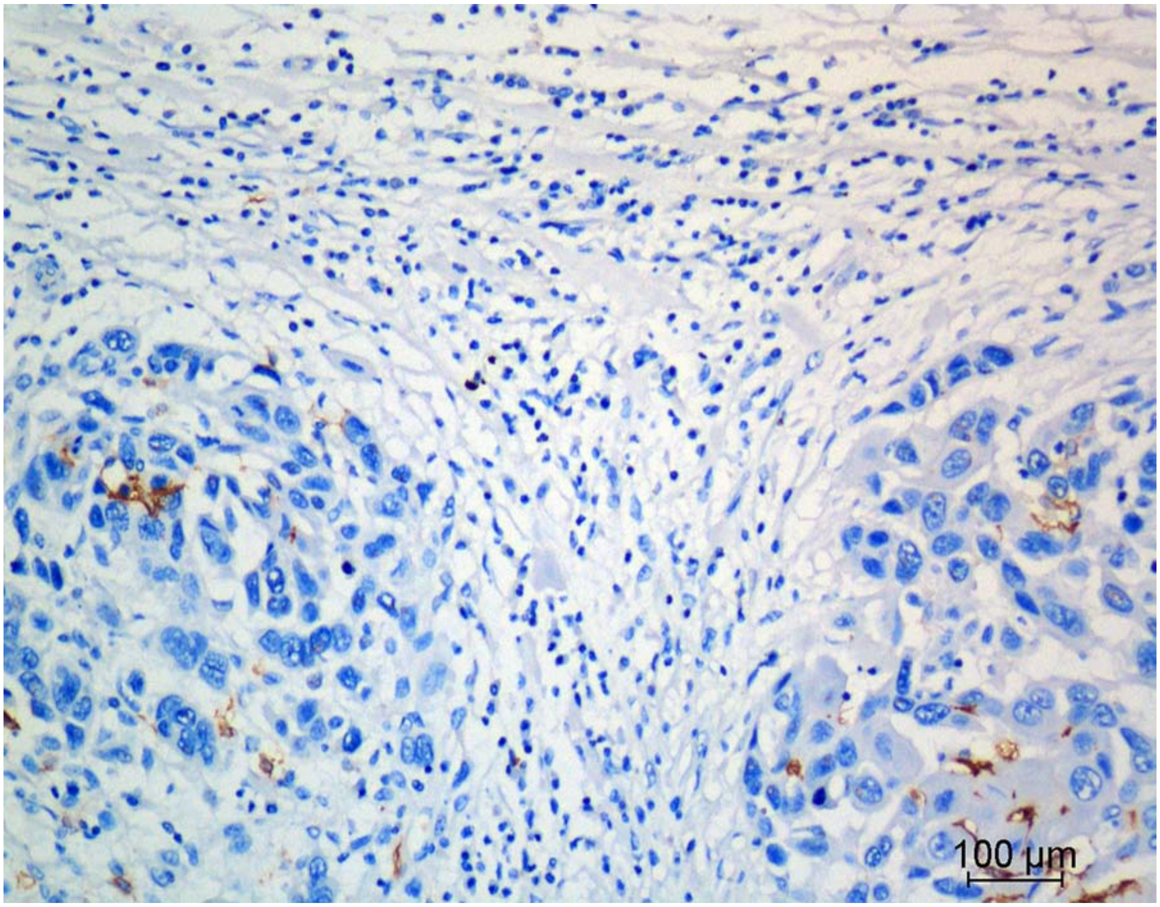

Supplementary Figure S1: IHC staining of CD1A in ESCC Tissue. Positive stained cells were demonstrated as brown.

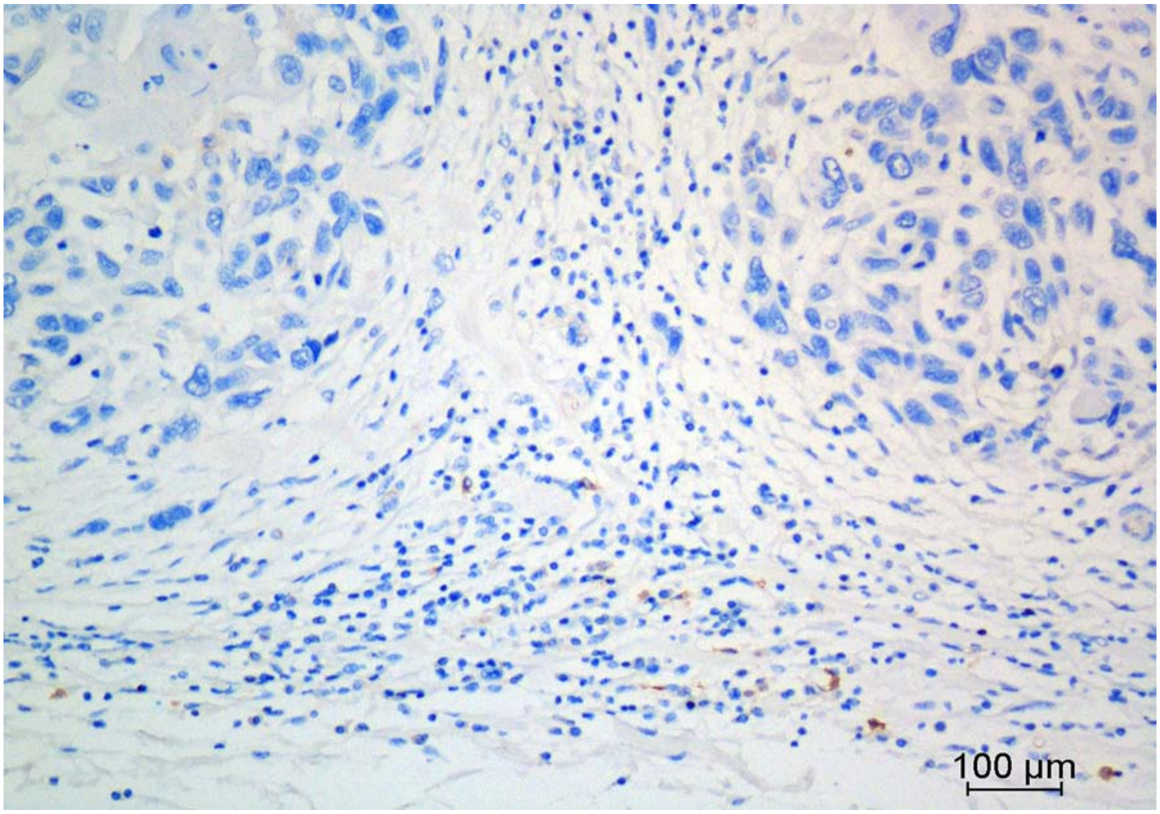

Supplementary Figure S2: IHC staining of CD123 in ESCC Tissue. Positive stained cells were demonstrated as brown.

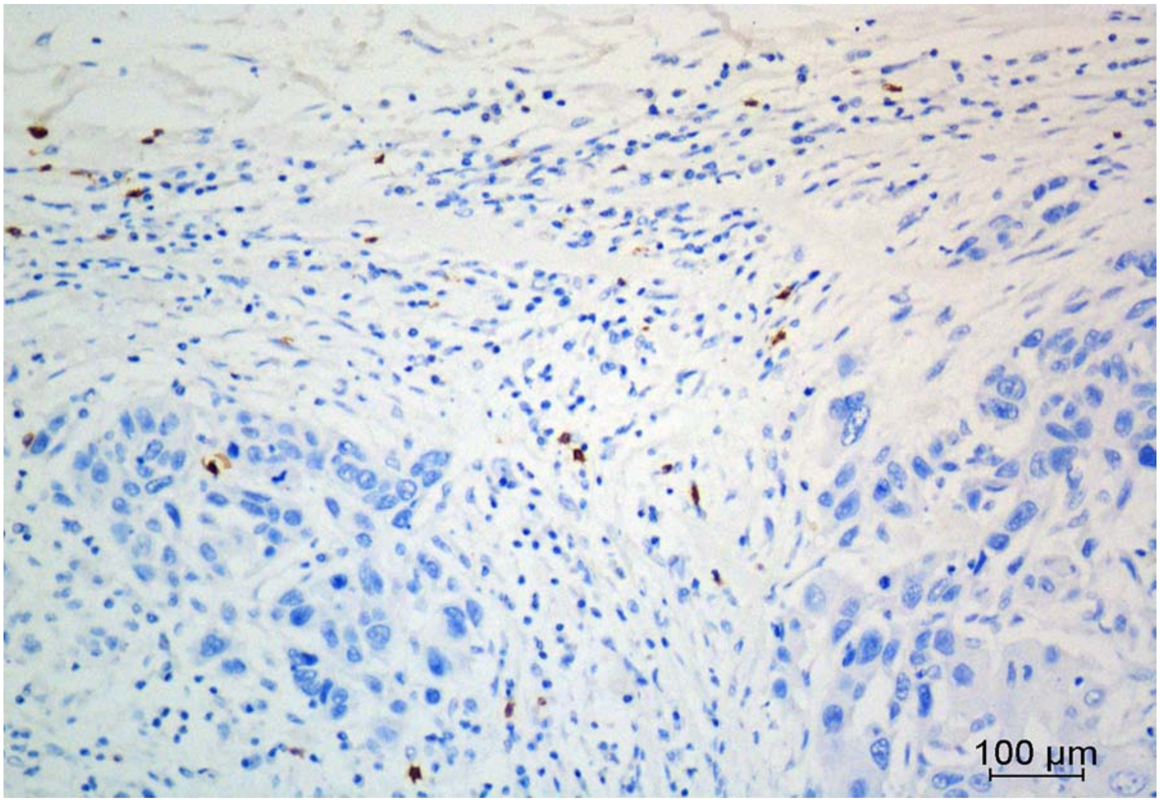

Supplementary Figure S3: IHC staining of CD57 in ESCC tissue. Positive stained cells were demonstrated as brown.

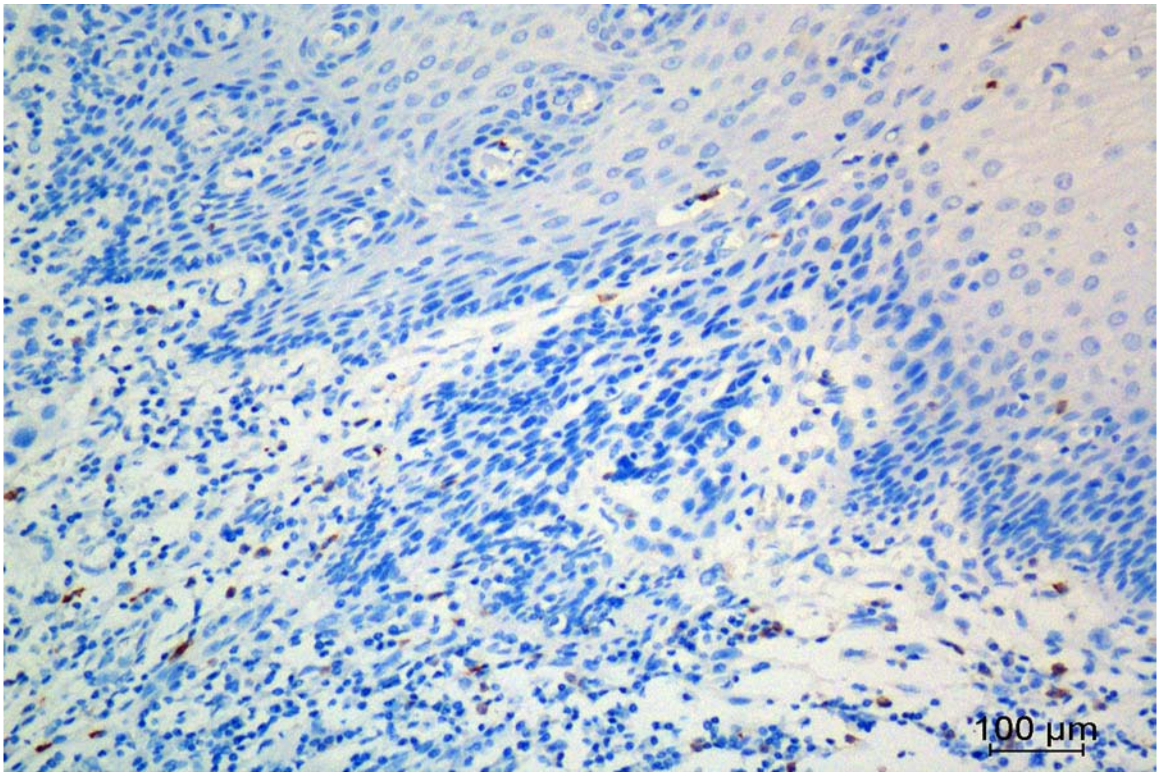

Supplementary Figure S4: IHC staining of CD66b in ESCC tissue. Positive stained cells were demonstrated as brown.

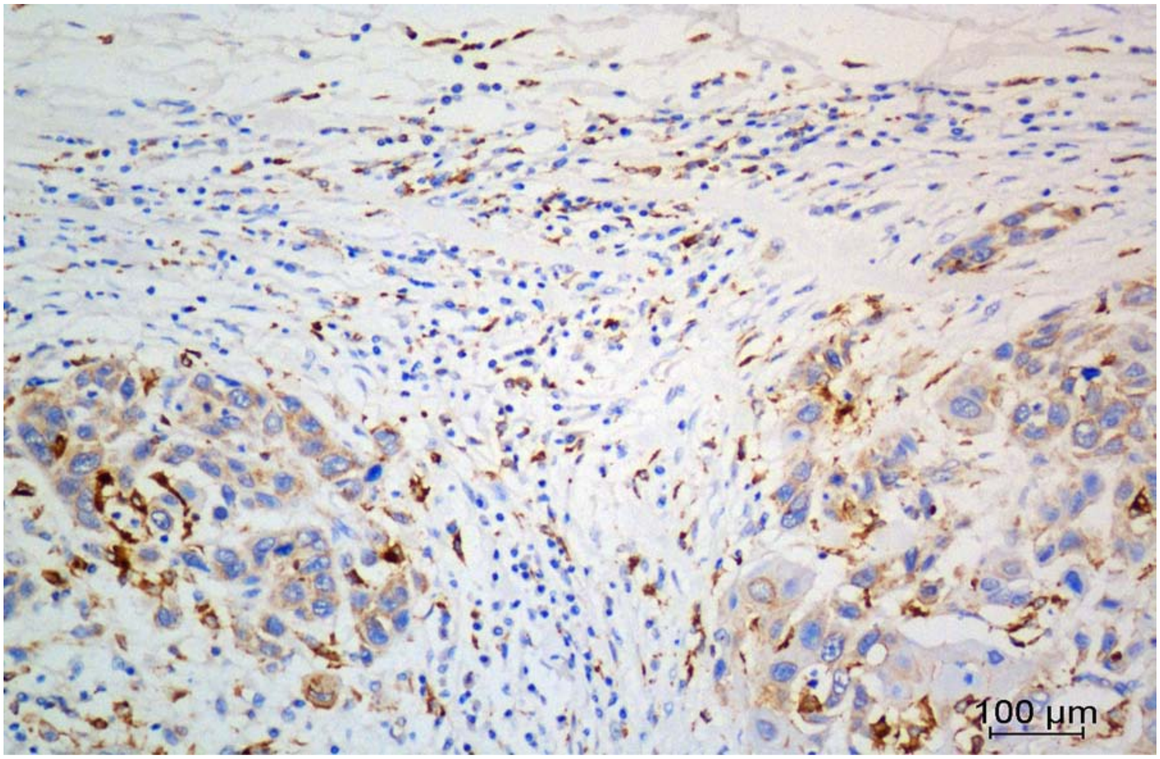

Supplementary Figure S5: IHC staining of CD68 in ESCC tissue. Positive stained cells were demonstrated as brown.

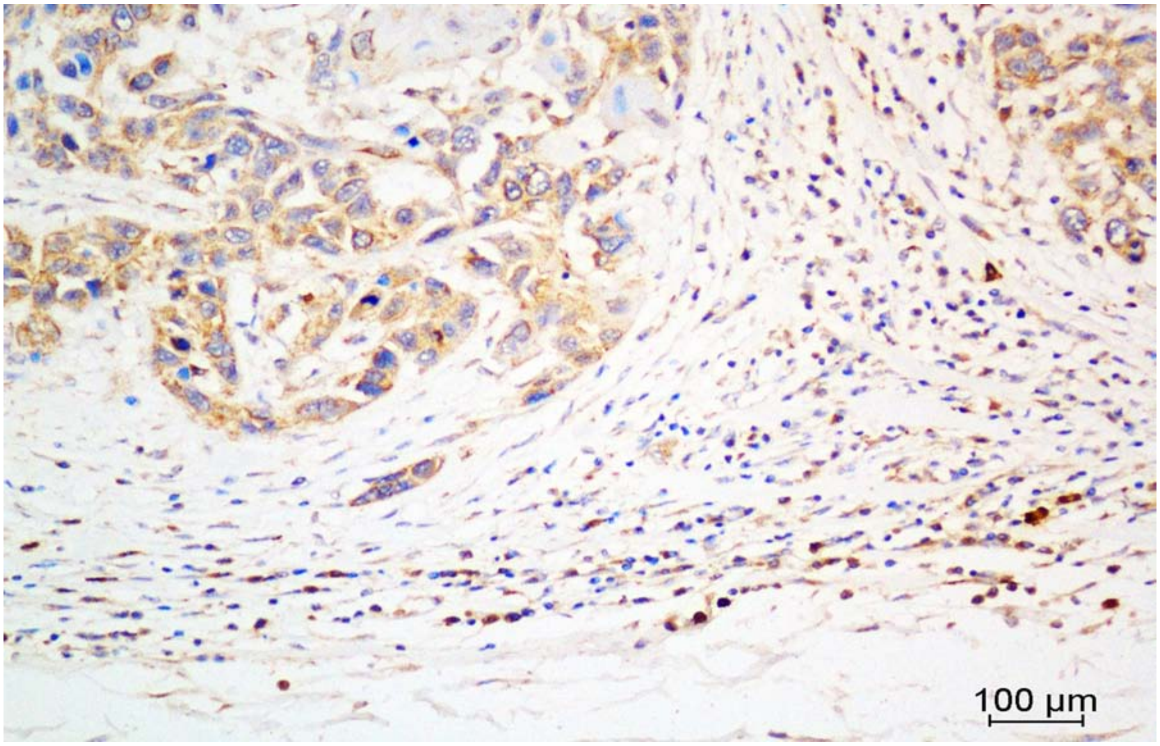

Supplementary Figure S6: IHC Staining of IL-13 in ESCC tissue. Positive stained cells were demonstrated as brown.

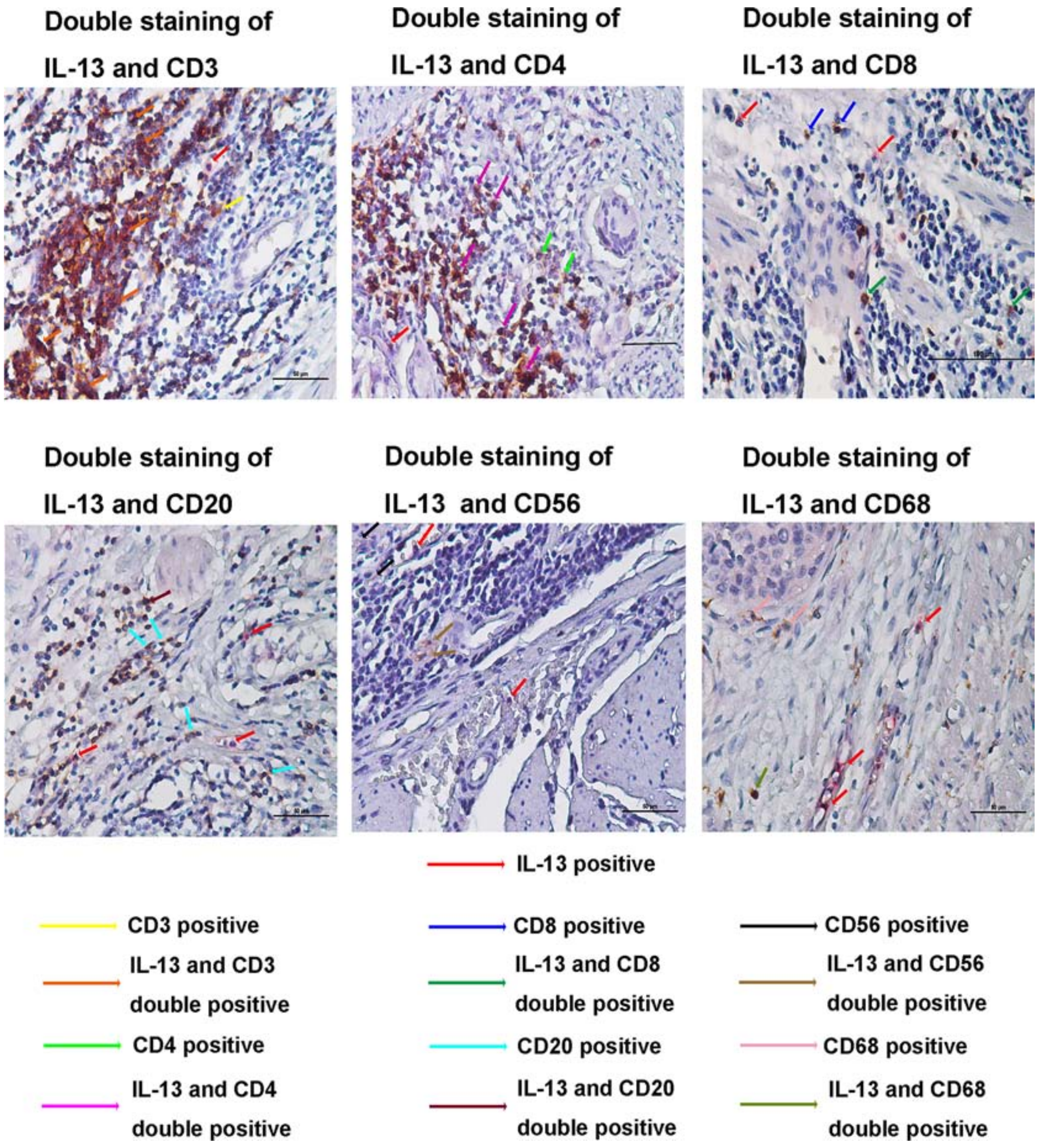

Supplementary Figure S7: Double IHC staining of IL-13 and indicated markers in ESCC tissue. IL-13 was stained as red and other CD molecular was stained as brown.
